# Rapid and Sensitive Detection of an Intracellular Pathogen in Human Peripheral Leukocytes with Hybridizing Magnetic Relaxation Nanosensors

**DOI:** 10.1371/journal.pone.0035326

**Published:** 2012-04-09

**Authors:** Charalambos Kaittanis, Hamza Boukhriss, Santimukul Santra, Saleh A. Naser, J. Manuel Perez

**Affiliations:** 1 NanoScience Technology Center, University of Central Florida, Orlando, Florida, United States of America; 2 Burnett School of Biomedical Sciences, College of Medicine, University of Central Florida, Orlando, Florida, United States of America; 3 Department of Chemistry, University of Central Florida, Orlando, Florida, United States of America; Naval Research Laboratory, United States of America

## Abstract

Bacterial infections are still a major global healthcare problem. The quick and sensitive detection of pathogens responsible for these infections would facilitate correct diagnosis of the disease and expedite treatment. Of major importance are intracellular slow-growing pathogens that reside within peripheral leukocytes, evading recognition by the immune system and detection by traditional culture methods. Herein, we report the use of hybridizing magnetic nanosensors (hMRS) for the detection of an intracellular pathogen, *Mycobacterium avium* spp. *paratuberculosis* (MAP). The hMRS are designed to bind to a unique genomic sequence found in the MAP genome, causing significant changes in the sample’s magnetic resonance signal. Clinically relevant samples, including tissue and blood, were screened with hMRS and results were compared with traditional PCR analysis. Within less than an hour, the hMRS identified MAP-positive samples in a library of laboratory cultures, clinical isolates, blood and homogenized tissues. Comparison of the hMRS with culture methods in terms of prediction of disease state revealed that the hMRS outperformed established culture methods, while being significantly faster (1 hour *vs* 12 weeks). Additionally, using a single instrument and one nanoparticle preparation we were able to detect the intracellular bacterial target in clinical samples at the genomic and epitope levels. Overall, since the nanoparticles are robust in diverse environmental settings and substantially more affordable than PCR enzymes, the potential clinical and field-based use of hMRS in the multiplexed identification of microbial pathogens and other disease-related biomarkers via a single, deployable instrument in clinical and complex environmental samples is foreseen.

## Introduction

Pathogenesis caused by intracellular pathogens, such as *Mycobacterium tuberculosis* among others, relies on the survival of the microorganism within host cells, such as macrophages and dendritic cells. This mode of infection with or without clinical symptomology hampers bacterial detection, preventing correct diagnosis. Consequently, the physician cannot proceed to the assignment of an appropriate treatment course, delaying the clearance of the pathogen from the body. Serological immunoassays cannot effectively detect intracellular pathogens in biological fluids (e.g. blood, lymphatic fluid) as the microorganism is hidden away within the immune cells. For this reason, isolation of the infected cells and extraction of their DNA, followed by detection of specific bacterial genomic markers via Polymerase Chain Reaction (PCR), is needed to facilitate identification of the pathogen. Although highly specific and sensitive, PCR methods are generally laborious, time-consuming and typically require homogeneous and pure DNA samples [1]. Evidently, complex biological samples, such as biopsies and blood, have to be thoroughly processed in multistep elaborate protocols to obtain pure DNA samples, significantly increasing the final readout time, and affecting the overall DNA yield. An alternative way to detect intracellular pathogens, particularly when they are in low numbers, is by isolating the infected leukocytes from the blood and expanding the number of viable bacteria via culturing methods before PCR analysis. Although very effective, bacterial culturing methods require a significant amount of time, such as in the case of slow-growing intracellular pathogens that have to be cultivated for even weeks, assuming that there is an adequate amount of viable pathogens in the clinical sample. Another drawback of culturing methods is that some pathogens do not grow effectively in culture, either because of the nature of the pathogen itself or the presence of biological interferences present in the clinical sample that prevent growth in culture. Because of these hurdles, new technologies that can detect the presence of a pathogen in human clinical samples are urgently needed.

It has been predicted that nanotechnology will have a major impact in medicine, agriculture and biotechnology among other fields [2], [3], [4], [5], [6], [7], [8], [9], [10]. Innovative technologies that take advantage of the unique size-dependent electronic, magnetic and luminescence properties that some nanomaterials exhibit when they specifically interact with a biological marker have been developed for faster and more accurate sensing of various pathogens [2], [3], [4], [5]. However, the translation of these technologies to the clinical diagnosis of an infectious disease and how it relates to the presence of the pathogen in clinical samples has been limited. The slow clinical translation of some of these nanotechnologies has been partly due to their poor performance in crude or minimally processed clinical samples. Among the most promising nanomaterials are magnetic relaxation nanosensors (MRS). These magnetic nanosensors are composed of a polymer-coated iron oxide nanoparticle onto which affinity ligands are conjugated to facilitate binding and magnetic detection of a particular target [11], [12]. Upon specific binding of a target to ligands on the magnetic nanoparticle, changes in the sample’s magnetic resonance signal (specifically the water proton relaxation time; T_2_) occur that correlate with the target concentration in solution. By measuring the changes in T_2_ relaxation times upon target interaction and correlating the intensity of the change with the target concentration, one can develop a sensitive detection method, therefore the acronym of magnetic relaxation nanosensors (MRS).

In contrast to other methods that utilize magnetic nanoparticles as labels and directly measure an intrinsic physical property, such as a magnetic field [13], [14], or a chemical property [6], the MRS method relies on the effect that the nanoparticle’s induced magnetic field exerts on the hundreds of thousands of water molecules surrounding the nanoparticle. This effect results in an amplification of the signal, allowing for sensitive detection even in turbid and minimally processed samples, without the need of further amplification, which is critical for assays such as PCR and ELISA. Hence, MRS-based assays can be more affordable than traditional assays, since they use a single reagent that can be easily produced in large quantities, and a single-step that avoids time- and labor-consuming procedures. Additionally, MRS can detect their targets at various point-of-care settings, since the nanosensors are stable at ambient conditions and utilize deployable instrumentation. We recently reported a unique MRS nanoparticle-target interaction that resulted in rapid T_2_ increases upon target binding [12]. This unique nanoparticle-target interaction is different from other MRS-based methods, because clustering of the nanoparticles is not required to achieve detection. In contrast, binding of MRS to the target caused fast, target-concentration-dependent increases in T_2_. Kinetic studies also revealed that the binding of MRS was faster and more sensitive than the MRS clustering, while achieving lower detection thresholds. In addition, nanoparticle valency – the amount of a targeting ligand on the surface of the nanoparticle – plays a role in the magnetic relaxation response. Nanoparticle valency controls the trend of the change in the spin-spin relaxation time (ΔΤ_2_). Specifically, it has been found that low valency results in decreases in the ΔΤ_2_ as the target concentration increases, while at high valency conditions the ΔΤ_2_ increases as the target concentration increases.

Hence considering the faster detection kinetics of MRS binding-based detection assays and the higher sensitivity of the MRS low-valency nanoparticle system, we developed hybridizing MRS (hMRS) that switched (increased) the sample’s water proton relaxation times (T_2_) upon binding to a unique bacterial genomic marker and tested their performance in clinical samples. The hMRS were designed to bind to a specific genomic marker in *Mycobacterium avium* spp. *paratuberculosis* (MAP); an intracellular pathogen known to cause Johne’s disease in cattle [15] and has been also implicated in the cause of Crohn’s disease in humans [16]. Furthermore, acknowledging the limitations of PCR in detecting nucleic acid biomarkers of intracellular pathogens in clinical samples, we investigated the hMRS performance in detecting MAP in human peripheral blood samples of Crohn’s disease patients as well as animal tissues with Johne’s disease.

MAP is found within the white blood cells of infected animals with Johne’s disease, a form of animal paratuberculosis, which is associated with chronic enteritis, reminiscent of Crohn’s disease in humans [16]. As early as 1913, Dalziel noted the clinical similarities of animal paratuberculosis, intestinal tuberculosis and human chronic granulomatous enteritis (Crohn’s disease) [17]. In humans, Crohn’s disease is a debilitating chronic inflammatory syndrome of the gastrointestinal track and adjacent lymph nodes [17], [18], [19]. The detection of MAP in tissues from patients with Crohn’s disease has been extensively reported. Of particular importance to our study is the report of the presence of MAP in human peripheral blood [20], [21], [22]. In these studies, MAP was identified by a culture method followed by PCR identification of a MAP genomic marker. The whole process took several months to complete, due to the slow growing nature of this pathogen. Such a slow detection method not only delays the diagnosis, but also slows any potential therapeutic intervention [20], [21]. Likewise, difficulties in detecting an intracellular pathogen, such as MAP, hamper studies aimed at the investigation of the potential role of MAP in Crohn’s disease pathology, as well as the pathogen’s impact on the dairy and beef industries.

Attracted by MAP’s slow growing characteristics, intracellular residence and clinical and agricultural relevance, we used hMRS for the identification of a highly conserved MAP genomic element (IS900). This unique DNA insertion sequence has been previously used to identify MAP in clinical samples and cultures by PCR, as it has not been found in other microorganisms or mycobacteria [23], [24], [25]. Detection is primarily achieved through either direct or culture-based nested PCR (nPCR), which use pure DNA extracts from infected leukocytes. Both PCR methods sequentially amplify a 398 bp fragment (amplification round 1) and a 298 bp internal sequence (amplification round 2). We reasoned that the binding of hMRS to IS900 would achieve faster and more sensitive detection of MAP DNA via magnetic relaxation in minimally processed clinical samples. Initial studies revealed that hMRS can specifically detect MAP’s genomic marker without showing cross-reactivity with other mycobacteria. Furthermore, the presence of MAP in homogenized tissues from Johne’s disease cattle was achieved at the genomic and epitope levels using hMRS and anti-MAP antibody-carrying MRS, demonstrating that the MRS technology can achieve detection at the genome and organism levels using diverse molecular probes. Finally, a library of 60 blood samples from Crohn’s disease patients and healthy individuals was screened with hMRS and nPCR assays. Using minimally processed blood samples, within 60 minutes the hMRS were able to detect the intracellular pathogen and better predict the clinical condition of an individual (healthy *vs* Crohn’s disease) than the nPCR methodologies. Specifically, hMRS outperformed the 12-week-long culture-based nPCR in clinical prognosis, as well as the least reliable direct nPCR, which are currently used in clinical and research studies. Overall, we show for the first time the use and validation of a novel translational nanotechnology, where the unique water relaxation properties of iron oxide nanoparticles and the dynamics of the nanoparticle/target interaction were harnessed to achieve a sensitive genomic biomarker detection system for an intracellular pathogen in samples directly obtained from human patients.

## Results

### Design and Synthesis of hMRS

**Figure 1:**
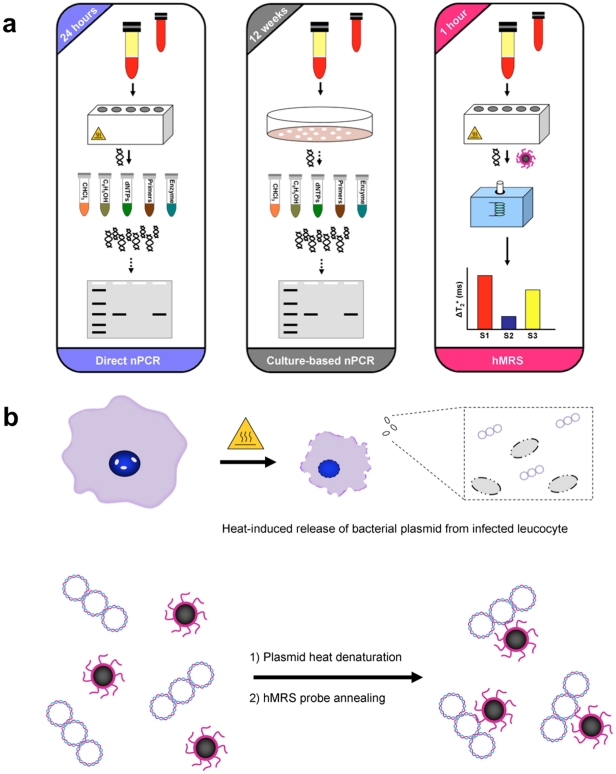
Design of a nanoparticle-based assay for the identification of genomic markers of the intracellular pathogen *Mycobacterium avium* spp. *paratuberculosis* (MAP). (**a**) Isolation of MAP requires collection of infected white bloods cells from blood samples via centrifugation. For direct nPCR analysis, DNA directly isolated from white blood cells is purified in multiple steps prior to amplification and detection by gel electrophoresis. Meanwhile, culture-based nPCR requires the growth of MAP in specialized liquid media for 12 weeks, followed by DNA isolation before nPCR. Hybridizing magnetic relaxation sensors (hMRS) can detect MAP DNA in minimally processed blood samples via changes in magnetic signal (ΔΤ2) in 1 hour, as opposed to 24 hours for direct nPCR and 12 weeks for culture nPCR. (**b**) Preparation of MAP DNA for hMRS. Heating of infected leucocytes facilities rupture of the cell membrane releasing MAP DNA. Further heating and cooling steps facilitates the annealing and binding of a MAP specific hMRS, resulting in an increase in the T_2_ water relaxation time.

The detection of MAP’s IS900 genomic marker relies on DNA isolation and subsequent amplification and detection via PCR. Typically, DNA from peripheral leukocytes found in the buffy coat (Direct nPCR) or leukocyte-derived cultured bacteria (Culture-based nPCR) is first isolated in a multistep protocol [21], yielding bacterial DNA of high quality (**Figure 1a**). However, this approach often times compromises DNA yield, which may impede MAP identification. In subsequent steps, the bacterial DNA is subjected to two 3-hour-long polymerase-mediated amplification rounds of the IS900 locus, followed by gel electrophoresis. To facilitate faster detection via magnetic relaxation, we designed a single hMRS probe that hybridizes with the unique IS900 sequence within the MAP plasmid. These hMRS are composed of a polyacrylic acid-coated iron oxide nanoparticle onto which an oligonucleotide sequence (ATGTGGTTGCTGTGT) complementary to the IS900 sequence in MAP is conjugated to facilitate binding and detection. The resulting MAP specific hMRS had a diameter of 78 ± 3 nm, zeta potential value of –35 mV, an average of 55 oligonucleotides per nanoparticle, with R1 and R2 relaxivities (at 0.47T) of 45 mM^-1^s^-1^ and 60 mM^-1^s^-1^ respectively. We anticipated that hMRS could utilize crude DNA, obtained from isolated buffy coats that have been subjected to heating (**Figure 1a**). It is well reported that heating of peripheral leukocytes accomplishes first the release of a pathogen from intracellular compartments [21], such as phagosomes, due to extensive pore formation on the host cell’s plasma membrane and organelles’ phospholipid-containing membrane. Once exposed to high temperatures, the pathogen’s structural integrity is affected, leading to release of its genomic DNA to the aqueous milieu. A series of heating and cooling procedures on the isolated MAP DNA plasmid in the presence of hMRS reduces the DNA torsional strain, facilitating annealing and hydridization of the hMRS onto the IS900 region of the MAP genome (**Figure 1b**). Decreasing the sample’s temperature can return the DNA to a higher structural order, with the hMRS still binding to the MAP genome and inducing increases in the sample’s magnetic resonance signal. Specifically, the hMRS were able to bind to MAP DNA after two short heating rounds (3 min each at 95°C), followed by unassisted cooling to room temperature. The fast changes in the magnetic resonance signal were monitored with a compact nuclear magnetic resonance instrument (0.47T, Bruker, Minispec), recording increases in the sample’s transverse relaxation times (ΔΤ_2_^+^, where T_2(sample)_ > T_2(control)_). Within 30 minutes after removing the samples from heat, significant and reproducible changes were observed prompting us to set this time-point as the hMRS readout point (**Figure S1**). Based on the observation that the sample’s T_2_ times were higher than those of the sterile control throughout the experimental time course, we deduced that the hMRS first bound to the relaxed circular MAP DNA that was heated, while the hMRS still remained bound to the cooled DNA as it was returning to its supercoil form. These results were corroborated by dynamic light scattering studies that showed no clustering of the nanoparticle suspension upon addition of the isolated MAP genome, since the nanoparticle diameter was found to be 81 ± 5 nm. Likely, this may be attributed to the fact that during sample cooling the DNA returned to its supercoiled conformation, which can be in the order of a few nanometers, and as a result nominally altering the overall size of the hybridizing nanoparticle – DNA complex.

### hMRS are Specific and Sensitive Probes

To determine the specificity of the hMRS, we incubated the hMRS (6 µg Fe/mL) with pure DNA extracts from various mycobacteria and monitored changes in T_2_ signal.

**Figure 2:**
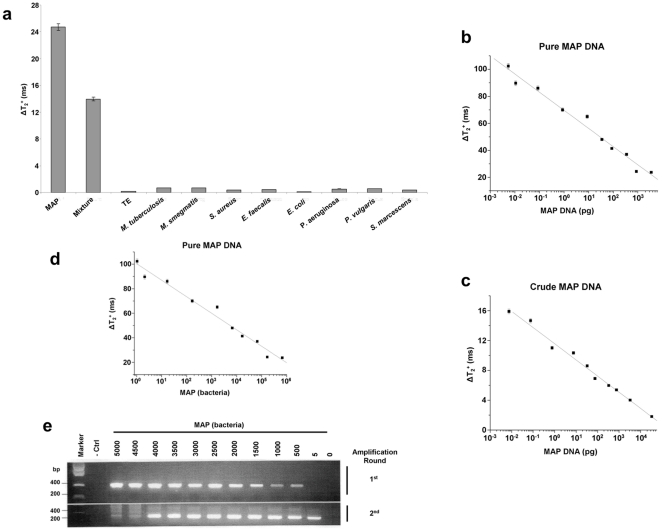
Specificity and sensitivity of the IS900-detecting hMRS. (**a**) hMRS specifically bind to MAP DNA and not DNA from other mycobacteria or common pathogens ([hMRS]  =  3 µg Fe/µL). hMRS facilitates the fast and quantitative detection of MAP DNA in pure (**b**) and crude (**c**) samples within 30 minutes. Comparison of the sensitivity of the hMRS and nested PCR (nPCR). (**d**) Within 30 minutes, the hMRS detected a single MAP genome copy, translating to one bacterium. (**e**) After its second amplification round (6 hours), nPCR achieved comparable sensitivity. (Upper gel image: first nPCR round (3 hours), lower gel image: second nPCR round (total two-round time 6 hours), - Ctrl: dH_2_O and 0: sterile TE buffer).

Within 30 minutes, results indicated that only samples containing MAP DNA exhibited a high ΔΤ_2_^+^ signal, being attributed to the hMRS – MAP DNA binding (**Figure 2a**). Additionally, the hMRS were still able to identify their target in a solution with a mixture of MAP and other mycobacterial DNA (**Figure 2a**). This indicates that the hMRS are sensitive and specific, without being affected by the presence of other mycobacterial DNA. Furthermore, addition of a synthetic oligonucleotide complementary to the IS900 sequence abrogated the signal of the hMRS upon MAP DNA addition, confirming the specificity of the hMRS probe (data not shown). Since other microorganisms’ DNA might be found in a clinical sample, we examined if the hMRS could differentiate between MAP and common Gram positive and Gram negative bacteria, as well as different types of mycobacteria. Results showed that only the MAP sample yielded a high ΔΤ_2_^+^, whereas the other bacterial DNA samples exhibited signal proximal to that of the sterile control (**Figure 2a**). These data hint the potential use of the hMRS and magnetic relaxation detection for the fast and specific detection of MAP DNA.

In subsequent studies, we determined if the hMRS could quantify MAP DNA. MAP DNA was isolated using either an elaborate multistep extraction protocol to obtain pure DNA, or a fast 30-minute boiling-based methodology to obtain crude DNA. After a 30-minute incubation period, the hMRS were able to detect MAP DNA in both pure and crude DNA preparations, exhibiting concentration-dependent ΔΤ_2_^+^ changes (**Figures 2b and 2c** **, Table S1**). The observed MAP concentration response pattern exhibited a high magnetic resonance signal (ΔΤ_2_^+^) at low target concentration levels and low signals at higher DNA concentrations. This suggests that the binding between the hMRS and MAP DNA was facilitated via low valency interaction, as previously described in other models [11]. This unique interaction mechanism provides enhanced sensitivity during the screening of scarce biomarkers, since high signal is desired at low target concentrations. Quantification of the number of oligonucleotides on the hMRS probe revealed that on average there were 55 oligonucleotides (oligos) per hMRS. This level of oligos per nanoparticle is in line with previous reports, where valency grafting of 46 oligos per nanoparticle exhibited a low valency binding behavior, whereas a nanoparticle with 368 oligos obeyed a high valency mechanism [12]. A low-valency-based detection approach is ideal for the identification of MAP in clinical samples, since it will induce a prominent ΔΤ_2_^+^ signal at low numbers of MAP. Based on the findings that both pure and crude MAP DNA exhibited similar hMRS quantification patterns, it was deduced that the hMRS assay can detect IS900 independent of the sample’s DNA extraction methodology. Although higher changes were recorded in pure MAP DNA samples, both extraction methodologies had equivalent detection thresholds, reaching low femtogram (10–^15^) levels. Potentially the differences in the magnitude of the ΔΤ_2_^+^ from pure and crude MAP DNA might be attributed to the sample’s unique characteristics. It is plausible that the high purity of the pure DNA extracts is associated with marginal protein and lipid levels, resulting in lower viscosity. Alternatively, a crude MAP DNA sample may have substantially higher content of proteins and lipids, thus higher viscosity, which may be reflected in lower ΔΤ_2_^+^ values upon IS9000 – hMRS hybridization We then examined whether nested PCR (nPCR) can achieve comparable sensitivity using crude MAP DNA. We decided to compare our hMRS method with nPCR as opposed to reverse transcriptase PCR (RT-PCR), due to the fact that MAP is an intracellular pathogen that grows very slowly. Similar to other intracellular microorganisms, it can be dormant and reduce its metabolism, suppressing transcriptional and translational activity [21]. As a result, the transcripts (mRNA) of even housekeeping genes are nominal and cannot be used in RT-PCR diagnostics. Therefore, nPCR is the technique most typically used for MAP detection [21]. Using nPCR, after two 3-hour-long cycles and electrophoresis, nPCR was not able to detect MAP IS900 in samples containing crude MAP DNA (**Figure S2**). This demonstrates the sensitivity and robustness of hMRS to rapidly detect their target even under conditions of interference, which prevent the use of traditional enzyme-based methods. In line with this, recent studies have demonstrated that nucleic-acid-decorated nanostructures can be highly stable even in serum-containing media and avoid non-specific protein and nuclease interactions, due to the steric inaccessibility and high local salt concentration introduced by the nanoparticles’ DNA strands [26]. Subsequently, we then compared hMRS and nPCR using purified DNA extracts instead. Also considering the size of MAP’s genome (4,829,781 bp) and the average molecular weight of a base pair (MW: 650), we calculated the mass of a single MAP genome copy to be equal to 5.2 fg. This value is comparable to that determined experimentally in previous reports [27]. Hence, we accordingly prepared serial dilutions of MAP DNA with known genome copy numbers. hMRS and magnetic relaxation detection were able to detect a single copy of MAP DNA within 30 minutes, whereas nPCR was able to achieve similar, although not as sensitive, detection (5 bacteria) only after the second amplification round (**Figure 2d and 2e** **, Table S2**). This translates to a total readout time for nPCR of more than 6 hours, with the need of more reagents, such as primers and enzymes, as well as labor and equipment time. Intriguingly though, the hMRS had a single genome-copy detection threshold when crude MAP DNA was used, without compromising their sensitivity in this minimally processed sample (**Figure S3**). Overall, these data demonstrate that hMRS can achieve fast single MAP genome detection even in crude DNA samples, outperforming nPCR in readout time, sensitivity and robustness.

### hMRS Achieve Genome-based Detection of the Intracellular Pathogen *Mycobacterium avium* spp. *Paratuberculosis* in Cultured Clinical Isolates and Tissue Samples

**Figure 3:**
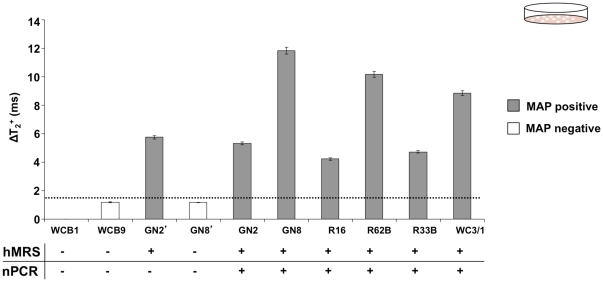
Screening of clinical isolates with hMRS and nPCR. Comparison between the magnetic relaxation-mediated detection of MAP in clinical isolates using hMRS after crude DNA extraction and culture-based nPCR. All isolates were from blood samples from Crohn’s disease patients, apart from GN2’ and GN8’ that correspond to ileal biopsies.

Since the hMRS detected MAP DNA in samples from pure bacterial cultures, we investigated if the nanosensors could identify their targets in more complex samples, such as cultured clinical isolates and homogenized tissue preparations. We first screened cultures of clinical isolates from Crohn’s disease patients with the hMRS and compared these results with nPCR. Specifically, we used crude DNA for the hMRS and pure DNA for the nPCR, since nPCR cannot utilize crude DNA. Following a single-step procedure, crude DNA obtained from cultured isolates of healthy individuals yielded a magnetic signal change that was comparable to that of the sterile culture medium (ΔΤ_2_^+^  =  1.3 ± 0.2 ms), establishing this magnetic signal change as the hMRS negative control baseline. Further studies indicated that the hMRS were able to rapidly detect MAP in several clinical isolates with good correlation to the culture-based nPCR, indicating that the hMRS can quickly detect the IS900 biomarker in minimally processed cultured clinical isolates (**Figure 3** **, Table S3**). Out of the ten cultures screened, one sample from a culture of an ileal biopsy (GN2’) was identified as positive by the hMRS, yet negative by nPCR. A false negative result with culturing methods can occur when either low amounts of the pathogen are present in the collected sample or when the pathogen is not viable to properly grow in culture. This prompted us to analyze the culture sample from the corresponding patient’s blood (GN2). We reasoned that viable MAP could be present in the patient’s leucocytes, granting correct diagnosis. Indeed, culture from the blood sample GN2 was identified as positive in agreement with the hMRS diagnosis and clinical sympthology. Therefore, nPCR may have failed to detect IS900 in ileal biopsy GN2’ perhaps due to either low levels of the pathogen or the presence of interferences in the biopsy sample that inhibited growth of the pathogen in culture. Overall, these results suggest that the high sensitivity of hMRS facilitates the positive identification of MAP in blood and biopsy samples, whereas PCR techniques were not always successful in identifying MAP.

**Figure 4:**
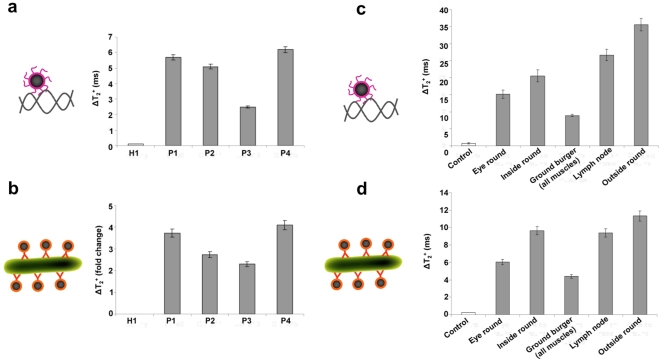
Comparison between the hMRS genomic detection of MAP in clinical isolates and homogenized tissues with the presence of MAP protein markers using anti-MAP antibodies conjugated MRS. (**a**) hMRS detected MAP genomic tags in clinical isolates via changes in the magnetic resonance signal. (**b**) A second preparation of magnetic nanosensors carrying polyclonal anti-MAP antibodies (anti-MAP-pAb) was able to corroborate the presence of MAP in these clinical isolates by the identification of MAP surface epitopes. (**c**) hMRS detected MAP’s IS900 in homogenized tissue from Johne’s disease cattle. (**d**) Corroboration of the presence of MAP in the cattle samples using anti-MAP-pAb MRS nanosensors.

Next, we screened various ileal biopsies from Crohn’s disease patients for the presence of MAP at the genomic and epitope levels using magnetic relaxation. We performed these studies since clinical diagnostics frequently require the identification of a biomarker at multiple levels. For instance, the aberrant activity of an oncogene has to be evaluated at the transcriptional, translational and post-translational levels, in order to understand causality, delineate disease dynamics and identify optimal therapy [28], [29], [30]. For these studies, in addition to the MAP IS900 hybridizing sensor (hMRS), we prepared a MAP-epitope-sensing MRS (diameter  =  92±4 nm, zeta potential  =  –31 mV, an average of 2 antibodies per nanoparticle, R_1_  =  48 mM^-1^s^-1^ and R_2_  =  57 mM^-1^s^-1^) that identifies a conserved MAP bacterial surface antigen [31]. Despite their different detecting mechanisms (oligonucleotide probe *vs* antibody), binding site topology and target characteristics (DNA *vs* surface epitope), both MRS provided a ΔT_2_^+^ signal for all samples with clinical symptomology (**P1-P4,** **Figure 4a and 4b**), whereas the sample from a healthy individual (**H1,** **Figure 4a and 4b**), exhibited nominal magnetic signal changes. Independent confirmation of the MAP MRS findings was achieved by performing DNA extraction and nPCR analysis. In addition, we used both the hMRS and epitope MRS to screen for the presence of MAP in tissue samples from cattle infected with Johne’s disease. Since MAP causes Johne’s disease in cattle, we investigated if these probes can identify this intracellular pathogen at the genomic and epitope levels in homogenized tissue samples. Results showed significant magnetic signal changes in all tissues from infected animals (**Figures 4c and 4d**), demonstrating that hMRS and epitope MRS can quickly detect MAP in clinically relevant samples at both the genomic and epitope levels. It is interesting to note that in both human biopsies from Crohn’s patients and tissues from animals with Johne’s disease, the magnetic relaxation signal obtained with the hMRS probe correlates with that obtained with the epitope-sensing MRS for the same sample. The differences in the ΔΤ_2_^+^ among the animal tissue samples might be attributed to the fact that the spatial concentration of MAP differs among the various tissues within the same animal due to Johne’s disease pathophysiology. Overall, these results indicate that these probes can be used for the screening of complex tissue samples with minimal or no sample preparation, supporting clinical decision making.

### hMRS Quickly Detect MAP DNA in Peripheral Blood

**Figure 5:**
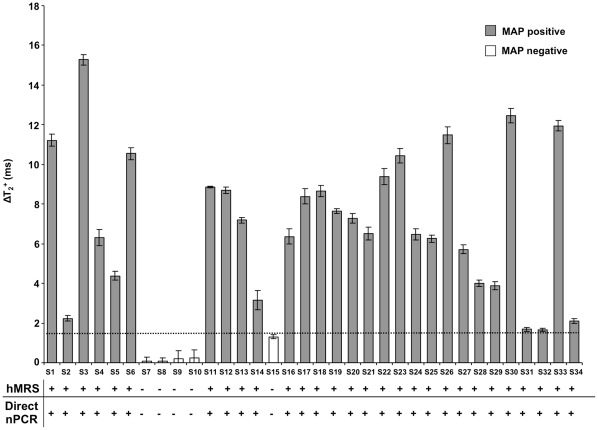
Detection of *Mycobacterium avium* spp *paratuberculosis*’ genomic marker in clinical samples with hMRS and direct nPCR. The hMRS detected MAP’s IS900 region in minimally processed blood samples (Cohort 1, n  =  34). Correlation of the hMRS findings was achieved through pure DNA extraction from white blood cells and direct nPCR. Results are means ± SE.

Acknowledging previous reports that identified MAP in the blood of Crohn’s disease patients, we sought to screen blood samples from healthy individuals and Crohn’s disease patients for the presence of IS900 (n = 34). For this, we chose to screen MAP’s IS900 genomic marker, rather than a protein (epitope) marker, since the later involves the use of anti-MAP antibodies that are difficult to reproducibly generate while avoiding antibody batch variabilities. In addition, a DNA-based hybridizing MRS would be more robust and selective than an antibody-based MRS. Peripheral blood was collected and DNA was directly isolated from white blood cells, via either a 30-minutes crude DNA extraction protocol for hMRS or a multistep high purity and quality DNA isolation procedure for direct nPCR screening. Direct nPCR for the MAP IS900 biomarker provided conclusive results about the pathogen’s presence in these samples, hence these samples established the first peripheral blood cohort (Cohort 1). In this cohort, positive samples were defined as those samples where a prominent band corresponding to IS900 was observed and negative samples were those where no visible band was observed at all. Control and Cohort 1 samples were screened with hMRS, after a 30-minutes crude DNA extraction protocol from white blood cells. Crude DNA extracted from control samples induced small changes in the magnetic signal (ΔΤ_2_^+^  =  1.3 ± 0.1 ms), which served as the hMRS reference point. Interestingly, results obtained with hMRS and magnetic relaxation detection using crude DNA samples from patients within Cohort 1 were in complete agreement with results obtained with direct nPCR that used pure DNA samples (**Figure 5** **, Table S4**). Addition of a synthetic target complementary to hMRS led to signal abrogation in the samples that had been identified as MAP positive, confirming the specificity of the assay. This indicates that the hMRS can detect their target in minimally processed blood samples, while eliminating lengthy sample preparation procedures.

**Figure 6:**
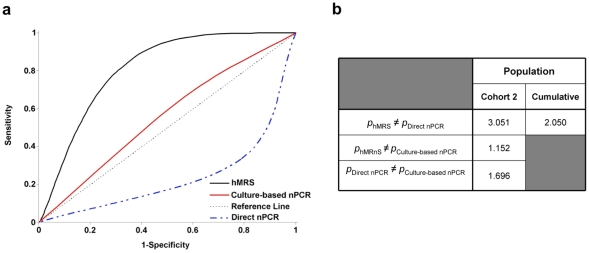
Detection of *Mycobacterium avium* spp *paratuberculosis*’ genomic marker in clinical samples with hMRS and culture-based PCR. (**a**) Blood samples from a second population (Cohort 2, n  =  26) were screened for the presence of the MAP IS900 genomic element with hMRS, direct nPCR and culture-based nPCR after 12-week cultivation. The receiver operating characteristic (ROC) curve indicated that the hMRS, can better differentiate between symptomatic (Chrohn’s) and asymptomatic (healthy) states than the two gold standard methods. (**b**) Two-proportion z-test confirmed hMRS as a better clinical diagnostic utility than the PCR-based methods.

Next, a second cohort of blood samples (Cohort 2, **Table 1**) was represented by samples where direct nPCR analysis provided dubious results. In samples within this cohort, classification as either positive or negative was not possible due to the presence of smeared or faint bands. The presence of these dubious samples, either false-positives or false-negatives, is a major problem with current nPCR techniques. Therefore, we examined if the hMRS performs better than direct nPCR and culture-based nPCR in determining if an individual is positive or negative based on his/her clinical state (in this case being Crohn’s positive or healthy). For the culture-based nPCR, inoculants from white blood cells were incubated for 12 weeks in specialized mycobacterial media (n = 26), allowing the potential growth of any viable MAP bacteria. After this long incubation period, pure DNA extraction was performed, followed by nPCR analysis. Interestingly, the culture-based nPCR was better in predicting disease than direct nPCR, which was performed on pure DNA extracted from white blood cells readily after venipuncture (blood draw) (**Figures 6a and 6b**). This may be attributed to the ability of bacterial culturing to provide more high-quality MAP DNA, as opposed to DNA directly obtained from white blood cells. Additionally, it is plausible that culturing can facilitate growth of even low populations of bacteria in optimal conditions, whereas direct pure DNA isolation from white blood cells may result in low genome recoveries of rare bacteria throughout its multiple steps and presence of interferences, like nucleases, cytosolic proteins and membrane lipids. Comparing both PCR setups with the crude DNA hMRS methodology, we found that the nanoparticle-based method outperformed both PCR-based methods in correlating MAP’s presence and disease state (**Figures 6a and 6b**). This hints the hMRS’ sensitivity, and the ability of the crude DNA extraction method to provide adequate DNA levels that are sufficient to facilitate improved bacterial detection. Furthermore, a receiver operating characteristic (ROC) analysis based on hMRS-mediated MAP quantification indicated that hMRS can provide improved clinical diagnosis than the qualitative (positive *vs* negative) nested PCR assays (**Figure 6a** **,** **Table 1**). Specifically, hMRS were able to better correlate bacterial biomarker presence and Crohn’s disease symptomology than both direct and culture-based nPCR. This suggests the potential use of hMRS for the future investigation of complex disease and the role of various disease effectors and contributors, to better understand pathogenesis’ mechanisms and its dynamic interactions. Taken together, this data prove that hMRS can rapidly detect a unique nucleic acid signature of an intracellular pathogen in clinical samples with improved fidelity and higher sensitivity than PCR. This enhanced detection translates into a more sensitive pathogen detection and more accurate diagnosis of the disease, providing important clinical information for successful treatment and therapeutic interventions due to early diagnosis.

**Table 1:** Clinical data and hMRS results of minimally processed blood samples (Cohort 2).

Sample	Age (years)/sex	Diagnosis	Direct PCR	Culture-based PCR	hMRS		
					MAP	ΔΤ2 (ms)	MAP (bacteria)
B1	F/51	CD	−	+	+ (3/3)	8.4	5348
B2	F/27	CD	−	+	+ (3/3)	8.6	4609
B3	F/73	Healthy	−	+	+ (3/3)	9.1	2542
B4	F/50	CD	−	+	+ (3/3)	3.4	1088531
B5	M/56	CD	−	+	+ (3/3)	7.6	12826
B6	F/57	Healthy	−	+	+ (3/3)	4.2	465264
B7	M/28	CD	−	+	+ (3/3)	7.5	13963
B8	F/31	Healthy	−	+	+ (3/3)	7.0	24006
B9	M/18	CD	−	+	+ (3/3)	10.3	760
B10	M/28	CD	−	+	+ (3/3)	5.2	162513
B11	F/16	CD	−	+	+ (3/3)	4.4	400959
B12	F/24	Healthy	+	−	− (0/3)	0.2	0
B13	F/61	Healthy	+	−	− (0/3)	0.4	0
B14	M/65	Healthy	+	−	− (0/3)	0.1	0
B15	F/62	Healthy	+	−	− (0/3)	0.1	0
B16	F/23	CD	+	−	− (0/3)	0.7	0
B17	F/48	Healthy	+	−	− (0/3)	0.6	0
B18	M/19	CD	+	−	− (0/3)	0.0	0
B19	F/26	Healthy	+	−	− (0/3)	0.4	0
B20	M/43	CD	+	−	− (0/3)	0.0	0
B21	F/50	CD	+	−	− (0/3)	0.1	0
B22	F/12	Healthy	+	+	− (0/3)	0.5	0
B23	M/57	Healthy	+	+	− (0/3)	0.0	0
B24	F/23	Healthy	+	+	− (0/3)	0.6	0
B25	M/61	Healthy	+	+	− (0/3)	0.2	0
B26	F/49	Healthy	+	+	− (0/3)	0.3	0

## Discussion

The primary aim of this study was to develop and test a nanoparticle-based assay for the sensitive detection of genomic bacterial biomarkers of an intracellular pathogen in clinical samples. Preliminary studies demonstrated that the hMRS can specifically detect a conserved genomic element (IS900) in MAP’s genome, and not in other microorganisms. Through additional studies, we identified that the sensitivity of the nanosensors was equal to one genome copy even in minimally processed samples, allowing detection of MAP in clinical isolates, tissue homogenates and blood. Compared to nPCR, hMRS had improved performance and yielded faster results. Consequently, this translates to lower costs, since the magnetic nanosensors are fairly easy and cheap to manufacture in larger quantities, and the assay does not require multiple oligonucleotide primer pairs, expensive enzymes and nucleotides. Detection was performed using a simple table-top relaxometer that measured the increase in the water proton relaxation time (NMR signal) that occurred after the hMRS probes bound to their genomic target. The unprecedented detection limit of this technique is due to the build-in water relaxation amplification that results upon a single binding event. The T_2_ relaxation times of hundreds of thousands of water molecules surrounding the hMRS probes increase upon binding of a single probe to the target. This is also facilitated by the low valency of the hMRS probe that induces drastic changes at low concentration of the target, or in this case low copy numbers of the genomic target. This approach contrasts with previous magnetic relaxation approaches that rely on the use of two magnetic nanoparticle probes that hybridize to adjacent sequences on a DNA target, facilitating clustering of the nanoparticles with a corresponding decrease in the T_2_ relaxation times [32], [33]. Since our approach does not rely on nanoparticle clustering and only the binding of the nanoparticle is sufficient for detection, a faster, more sensitive magnetic relaxation assay that rivals PCR has been developed for the screening of an intracellular pathogen genomic marker. Apart from a table-top relaxometer, portable magnetic relaxometers have been developed, suitable for point-of-care screening with hMRS [34].

hMRS can be used for the detection of diverse targets in complex media, including turbid and opaque clinical samples [11], [12]. Herein, our results demonstrate that the hMRS binding can achieve the unprecedented and fast detection of a single genome copy of an intracellular pathogen. This is critical for the identification of extremely virulent pathogens. As MAP is an intracellular pathogen similar to *M. tuberculosis*, its detection is difficult, due to the pathogen’s residence within an infected leukocyte’s organelles. Thus contemporary diagnostic methodologies rely on cell isolation, cell lysis and nucleic acid isolation, in order to yield high purity DNA. This renders MAP’s detection problematic since the microorganism has to be isolated from highly complex samples, such as blood or severely inflamed tissue biopsies, before amplification and detection via PCR analysis. Apart from DNA quality, the quantity of MAP DNA can affect the outcome of PCR. For instance, low MAP DNA levels from a clinical sample may not be successfully amplified through direct nPCR or biological matrix components can affect the polymerase activity, yielding false-negative or dubious results. In our studies, a significant number of samples that were identified as MAP dubious by direct nPCR were found to be positive by culture-based nPCR and hMRS (Cohort 2), indicating that the pathogen was originally present in the patient’s blood (albeit in low amounts) and successfully grew in culture at adequate levels.

The culture-based nPCR, relies on the availability of viable bacteria in the clinical sample that can be grown in culture in sufficient amounts before nPCR. Most clinical microbial diagnostics rely on the growth of the pathogen in culture, before performing the appropriate immunological or PCR tests [35], [36]. Growth can be observed within 12 to 24 hours for fast-growing microorganisms, but for slow-growing intracellular microorganisms like MAP it can take from a week up to several months. Another problem with this technique is that some microorganisms are difficult to culture in the laboratory, which limits their detection. Although highly reliable, culture-based PCR can result in the identification of samples as false-negative, due to the absence of a viable pathogen or the inability of the pathogen to grow in culture. Therefore, it is plausible that samples that might be found to be positive by direct nPCR and hMRS to be identified as negative 12 weeks after by culture-based nPCR. This is common for low bacterial loads of slow-growing microorganisms that require highly specialized media and cultural conditions, whose inadequacy might drastically affect bacterial viability leading to cell death and DNA degradation [37]. These problems in detection with PCR methods are encountered in the detection of any intracellular pathogen, as the pathogen has optimized its adaptation and growth within a host cell. Due to their sensitivity and minimal sample processing, hMRS are capable of circumventing PCR’s obstacles and achieving improved clinical diagnosis. Our findings indicate that hMRS can detect an intracellular pathogen’s genomic marker in biological fluids (i.e. blood) and tissue homogenates within one hour, where the first 30 minutes are spent on crude DNA extraction and the remaining time on readout.

Herein, we have also demonstrated that MAP DNA can be detected in various tissues from Johne’s disease animals, as well as in biopsies and blood from Crohn’s disease patients, at the epitope and genomic biomarker levels. Furthermore, our results indicate that hMRS can detect the presence of MAP in Crohn’s disease patients more reliably than direct or cultured-based nPCR. As a matter of fact, hMRS is a better predictor of the state of the disease, correlating the clinical state of a patient with the presence of MAP in blood samples (**Figure 6**). The use of hMRS could be a powerful new tool in the study of the mechanism of infection of intracellular pathogens and its relation to disease state, as well as in elucidating intracellular pathogens’ adaptation strategies [38]. Difficulties in detecting MAP hamper studies aimed at the investigation of the potential role of MAP in Crohn’s disease pathology, as well as the pathogen’s impact on the dairy and beef industries [39]. Overall, these studies provide evidence for the use of hybridizing magnetic nanosensors and magnetic relaxation detection to reliably identify the presence of an intracellular pathogen in clinical samples in a fast, cost-effective and highly sensitive way.

## Materials and Methods

### Preparation of IS900-specific hMRS

The IS900-specific hMRS were prepared from propargylated polyacrylic-acid-coated iron oxide nanoparticles, utilizing literature available protocols [11]. Specifically, incorporation of propargyl groups on polyacrylic-acid-coated iron oxide nanoparticles was achieved via the carbodiimide chemistry, followed by magnetic separation of the nanoparticles, using an LS25 MACS column (Miltenyi) [11]. Subsequently, the propargyl-functionalized iron oxide nanoparticles were reacted with an azide-modified oligonucleotide, which is complementary to a segment of MAP’s IS900 genomic element. Conjugation of the azide-terminated 15-bp oligonucleotide 5′-ATGTGGTTGCTGTGT-3′ was achieved via “click” chemistry, as previously described [40]. Briefly, 400 µL propargylated iron oxide nanoparticles were resuspended in 1,100 µL NaHCO_3_ buffer (0.1 M, pH 8.4). To this, 200 µL of 10 mM TCEP (tris(2-carboxyethyl)phosphane hydrochloride, Sigma) were added as a reducing agent. Twenty µL of 6.3 M oligo were diluted in 80 µL NaHCO_3_ buffer (0.1 M, pH 8.4) and added to the nanoparticle solution. The reaction was initiated with the dropwise addition of 150 µL Cu(I)-TBTA complex (tris(benzyltriazolylmethyl) amine, 10 mM, Sigma), which was previously prepared in DI water and ^t^butanol (9∶1). The reaction was incubated at room temperature under continuous mixing for 3 hours, followed by overnight incubation at 4°C under constant mixing. The resulting IS900 hMRS were dialyzed against DI water using a 6,000 – 8,000 MWCO membrane (Spectrum), followed by magnetic separation with an LS25 MACS column (Miltenyi). The epitope-sensing MRS were formulated as previously reported, and the MAP antibody was obtained from Dr. Naser’s lab [12]. Briefly, polyacrylic-acid-coated iron oxide nanoparticles were conjugated to Protein G via the EDC/NHS chemistry, in order to have Protein G as a high-affinity immunoglobulin linker that provides optimal antibody orientation [12]. The reaction was performed according to the literature, followed by magnetic separation through a 1X-PBS-equilibrated LS25 column and antibody conjugation [12]. The nanoparticle valency of the antibody-carrying nanoparticles was assessed through quantification of the nanoparticles’ protein content, using published methodologies [11]. Size determination of all hMRS was achieved through dynamic light scattering, using the PDDLS CoolBatch 40T instrument and Precision Deconvolve 32 software. Zeta potential measurements were performed on a Malvern zetasizer, while the R_1_ and R_2_ relaxivities were determined after determination of the nanoparticle’s iron content and relaxation studies on a compact relaxometer (Minispec, Bruker). Determination of the hMRS oligonucleotide concentration was achieved by monitoring the absorbance at 260 and 305 nm (background), using a Nanodrop 1000 spectrophotometer (Thermo Scientific), as previously described [12]. The hMRS were stored at 4°C until further use.

### Bacterial Cultures, Isolates and Homogenized Tissue

Lab strains of MAP and clinical isolates were grown in 12B* BACTEC bottles (Becton Dickinson) as previously described [21]. Quantification of MAP grown in the BACTEC bottles was assessed with the BACTEC 460 TB Analyzer (Becton Dickinson). Heat-inactivation of MAP was performed by autoclaving the BACTEC bottle for 10 mins. *Proteus vulgaris* # 8427, *Staphylococcus aureus* #33862, *Pseudomonas aeruginosa* # 27853, *Enterococcus faecalis*, *Escherichia coli* # 8739, and *Serratia marcensis* were grown in culture tubes with nutrient broth, and bacterial growth was monitored spectrophotometrically. Heat inactivation of these bacteria was performed by autoclaving the culture tubes for 10 minutes. Upon inactivation, all bacterial stocks were placed in a Fisher Isotemp freezer (Fisher Scientific), until further use. Clinical isolates and tissue specimens from animals with Johne’s disease were obtained from tissue collections stored in Dr. Naser’s laboratory.

### Extraction of Pure and Crude Bacterial DNA from Bacterial Cultures

DNA was extracted from cultured bacteria in a class II biosafety cabinet, according to literature-available procedures [21]. One mL of bacterial cultures was aseptically transferred to microcentrifuge tubes, followed by centrifugation at 13,200 rpm. The resulting pellets were resuspended in 120 µL sterile TE buffer (10 mM Tris, 1 mM EDTA, pH 8.0) and incubated in a dry heat bath at 100°C. Samples without further processing were used as crude DNA extracts in MRnS and nPCR studies. In order to obtain pure bacterial DNA, after heating the samples, we placed them on ice for 15 minutes and then centrifuged them at 12,000 rpm for 10 minutes at 4°C. The supernatants were transferred to Phase-lock gel tubes (Eppendorf), which were supplemented with 200 µL of phenol/chloroform/isoamyl alcohol (1:1:24 v:v, Acros). The tubes were centrifuged for 5 minutes (12,000 rpm, 4°C), and the supernatants were precipitated using 400 µL 100% ethanol cooled to −20°C. The precipitated DNA was washed, dried, and finally reconstituted in 50 µL TE buffer. Pure and crude bacterial DNA was stored at 4°C until further analysis, whereas spectrophotometric quantification of DNA content was achieved using a compact spectrophotometer (Nanodrop 1000, Thermo Scientific). Crude and pure DNA extractions from clinical isolates were similarly performed.

### hMRS and Nested PCR Experiments

Serial dilutions of bacterial DNA were prepared in TE buffer. For the relaxation-mediated experiments, 200 µL of the nanoparticle suspension (6 µg Fe/mL in 0.1 M phosphate buffer, 0.1 M NaCl, pH 7.4) were incubated with 1 µL of bacterial DNA or control (TE buffer). The samples were heated twice for 3 minutes at 95°C. After this, the samples were transferred to relaxometer tubes and cooled down at room temperature, while taking relaxation measurements on a magnetic relaxometer (Minispec, Bruker). For the specificity assays using a complementary synthetic target (5′-TCCTTCGGCCATCCAACACAGCAACCACAT-3′), 1 µL of the target (1.1 M) was added to the hMRS mycobacterial samples containing the same DNA concentration (3.5 ng/µL). Similar DNA concentration was used for the specificity experiment utilizing other Gram-positive and Gram-negative bacteria and hMRS (3 µg Fe/mL). For the sensitivity experiments, serial dilutions of pure and crude MAP DNA were prepared from pure cultures of MAP grown in 12B* BACTEC bottles, which had been processed and quantified as described in the preceding section (DNA extraction methodology). Nested PCR (nPCR) was performed as previously described [21].

### Blood Samples, Extraction of Bacterial DNA from White Blood Cells and MAP Culturing

Blood samples were collected from healthy individuals and patients with Crohn’s disease at the University of Florida, College of Medicine, in accordance to Institutional Review Board guidelines. The samples were coded and sent to the University of Central Florida for blind screening via PCR and hMRS analyses, without knowing in advance the individual’s condition, demographics and MAP’s presence (Cohorts 1 and 2). Isolation of white blood cells was achieved via centrifugation at 3,000 rpm for 10 minutes at room temperature, as previously described [21]. Pure DNA was extracted via phenol/chloroform/isoamyl alcohol precipitation, similar to previously published methodologies. The pure DNA was subjected to direct nested PCR, as described above. Crude DNA extraction from the isolated white blood cells was achieved via centrifugation at 13,200 rpm for 2 minutes. The resulting pellets were resuspended in 120 µL sterile TE buffer (10 mM Tris, 1 mM EDTA, pH 8.0) and incubated in a dry heat bath at 100°C for 30 minutes, followed by cooling and centrifugation at 12,000 rpm that provided us with supernatants rich in crude DNA. For bacterial culturing, buffy coat samples were prepared in sterile phosphate buffered saline as described before and inoculated on MGIT bottles. The inoculants were first incubated at 37°C in a 5% CO_2_ atmosphere for 12 weeks, and then pure DNA extraction was performed as stated above.

### Measurement of Proton Relaxation Times

Spin-spin relaxation times (T_2_) were measured using a 0.47 T mq20 NMR analyzer (Minispec, Bruker). T_2_ values were obtained before and after addition of the sample, and through the time course of the study. All T2 measurements were performed using a CPMG pulse-echo train with a 1.5 ms interpulse spacing (Bruker Corp., Billerica, MA). The change in magnetic resonance signal (ΔΤ_2_^+^) was defined as the sample’s T_2_ minus the magnetic signal of the corresponding negative control. All experiments and measurements were carried out in triplicate and data were expressed as mean ± standard error, unless otherwise denoted.

### Statistical Analysis

Clinical diagnosis and sample collection was performed at the College of Medicine, University of Florida, based on clinical and endoscopic criteria [21]. Samples identified as IS900-positive by the hMRS method had an average ΔΤ_2_^+^ higher than 1.5 ms, while positive samples by the nested PCR method had a unique 298-bp nucleotide sequence. The averages of three independent experiments are reported, unless otherwise stated. Two-proportion z-test statistics were determined through the SPSS package, with the confidence level set at 95% (IBM Co.). ROC analysis was performed based on hMRS determination of the sample’s MAP load. This was achieved through the correlation of the ΔΤ_2_^+^ signal to bacterial genome copies, using a crude extracted DNA standard curve (**Figure S3**). For nPCR, the presence of a band corresponding to IS900 was marked as positive (value = 1), whereas its absence was assigned as negative (value = 0). The overall ROC methodology was performed as previously reported in literature [34]. Specifically, the classification variable was the individual’s clinical condition (Crohn’s disease *vs* healthy), whereas the output curve was fitted using logistic regression.

### Ethics Statement

The use of human subjects was approved by the University of Florida’s Institutional Review Board (protocol number 354–2007). A signed individual written informed consent agreement was obtained from each subject or in the case of children from their parent or legally authorized representative before enrolment in the study.

## Supporting Information

Figure S1 **Kinetics for MAP’s IS900 genomic marker detection with hMRS.** After heating the samples to facilitate DNA stand separation and hMRS hybridization, the changes in the T_2_ magnetic resonance signal were recorded over time, with marked changes occurring within less than an hour (Means±SE).
(TIF)

Figure S2 **Nested PCR (nPCR) cannot quantify crude MAP DNA.** – Ctrl: negative control (dH2O) of the first nPCR round, – Ctrl: negative control (dH2O) of the second nPCR round, TE: TE buffer, + Ctrl: two controls of pure extracted MAP DNA.
(TIF)

Figure S3 **Genome-copy-based quantification of MAP with hMRS and crude extracted DNA.** Samples with known amounts of crude DNA from cultured MAP were utilized to correlate the changes in the T2 signal (ΔT2+) and the number of bacteria originally present in the sample, using the MAP’s genome size as a reference. (Means±SE. SE too small to depict in high bacterial levels.)
(TIF)

Table S1 **Spin-spin relaxation times (T2) of crude MAP DNA samples.** Serial dilutions of crude MAP DNA samples have been utilized to assess the sensitivity of the hMRS method in minimally processed bacterial cultures. The averages of each independent experiment are listed and the studies’ mean.
(PDF)

Table S2 **Spin-spin relaxation times (T2) of pure MAP DNA samples with known genome copies.** Three independent experiments were performed on pure DNA samples obtained from cultured MAP. Correlation between DNA levels and bacterial populations was achieved by quantifying DNA spectrophotometrically and using the MAP genome size as a reference.
(PDF)

Table S3 **Demographics of cultured clinical isolates that were screened with hMRS and nPCR.** (CD: Crohn’s disease, IBD: inflammatory bowel disease)
(PDF)

Table S4 **Clinical data and hMRS results of Cohort 1 blood samples.** Samples from Crohn’s disease (CD) patients, healthy individuals or asymptomatic carriers were analyzed by direct nPCR and hMRS. Quantification of the MAP genome copies was achieved in positive (+) samples using a training standard curve for crude extracted MAP DNA.
(PDF)
