# Supplementary figures and images for: Rapid and Sensitive Detection of an Intracellular Pathogen in Human Peripheral Leukocytes with Hybridizing Magnetic Relaxation Nanosensors
Source: PLoS One. 2012 Apr 9;7(4):e35326. doi: 10.1371/journal.pone.0035326 (PMC3322147; doi:10.1371/journal.pone.0035326)

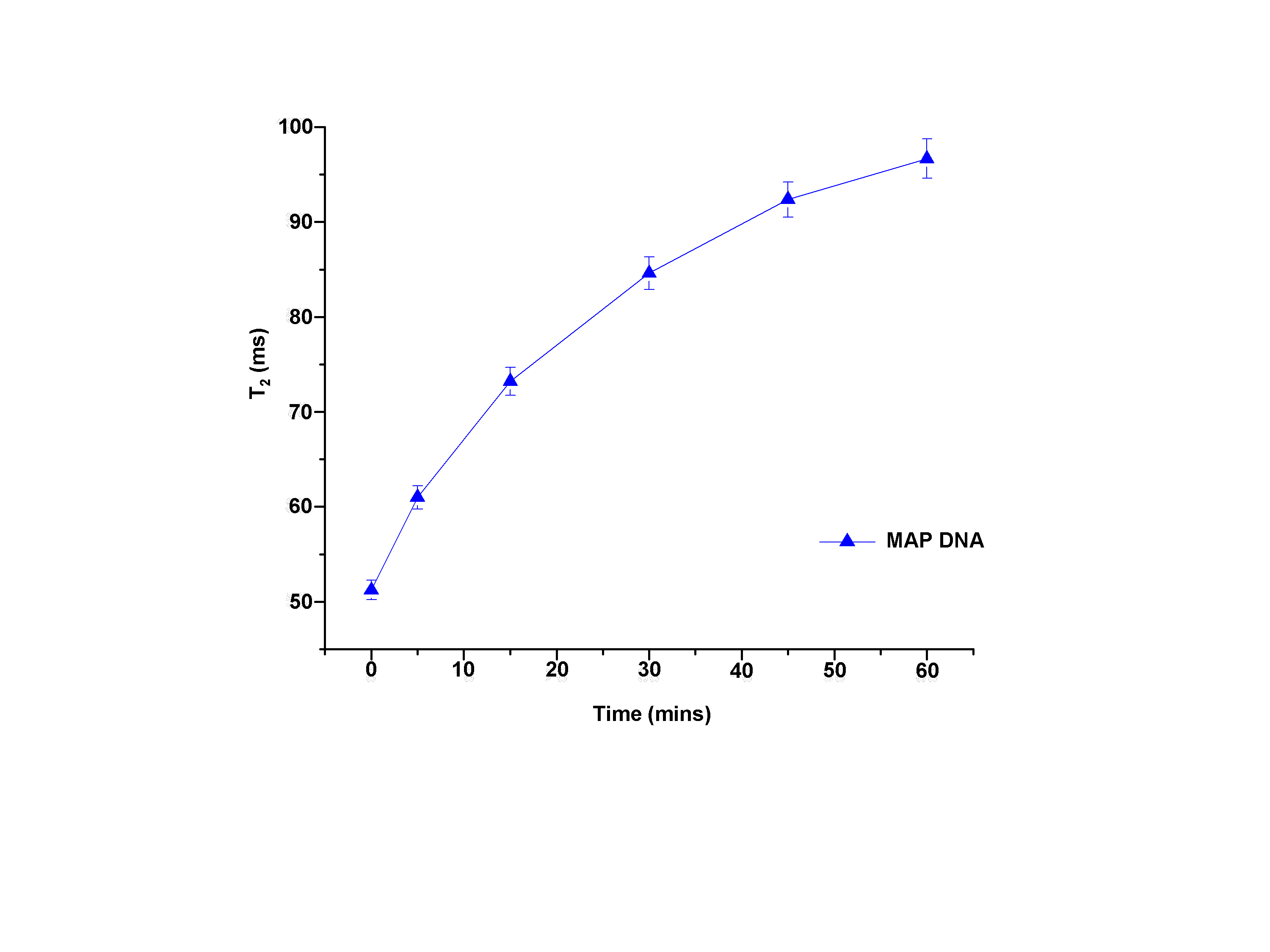

Supplement: Figure S1 — Kinetics for MAP’s IS900 genomic marker detection with hMRS. After heating the samples to facilitate DNA stand separation and hMRS hybridization, the changes in the T2 magnetic resonance signal were recorded over time, with marked changes occurring within less than an hour (Means±SE). (TIF) [file pone.0035326.s001.tif]

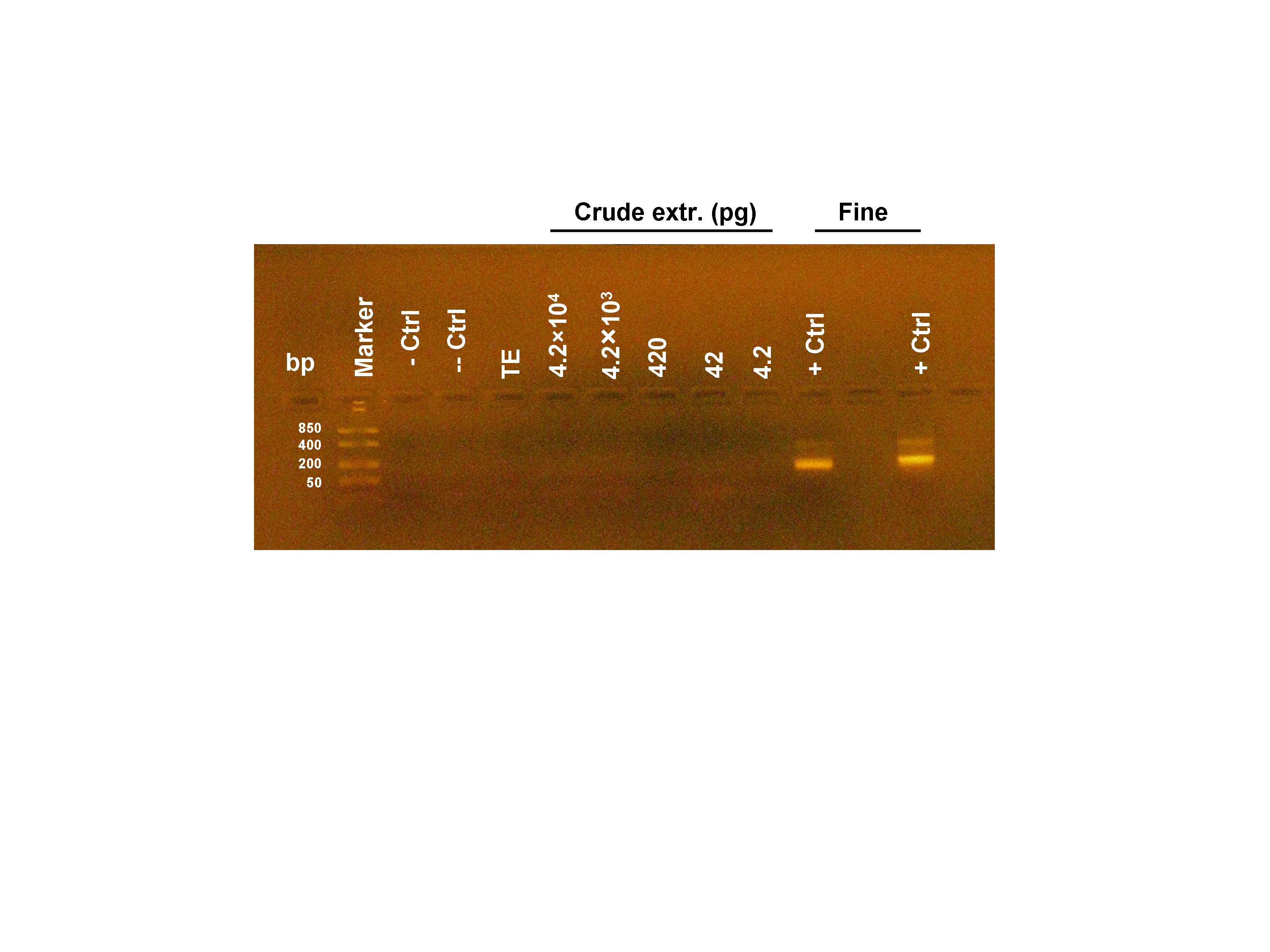

Supplement: Figure S2 — Nested PCR (nPCR) cannot quantify crude MAP DNA. – Ctrl: negative control (dH2O) of the first nPCR round, – Ctrl: negative control (dH2O) of the second nPCR round, TE: TE buffer, + Ctrl: two controls of pure extracted MAP DNA. (TIF) [file pone.0035326.s002.tif]

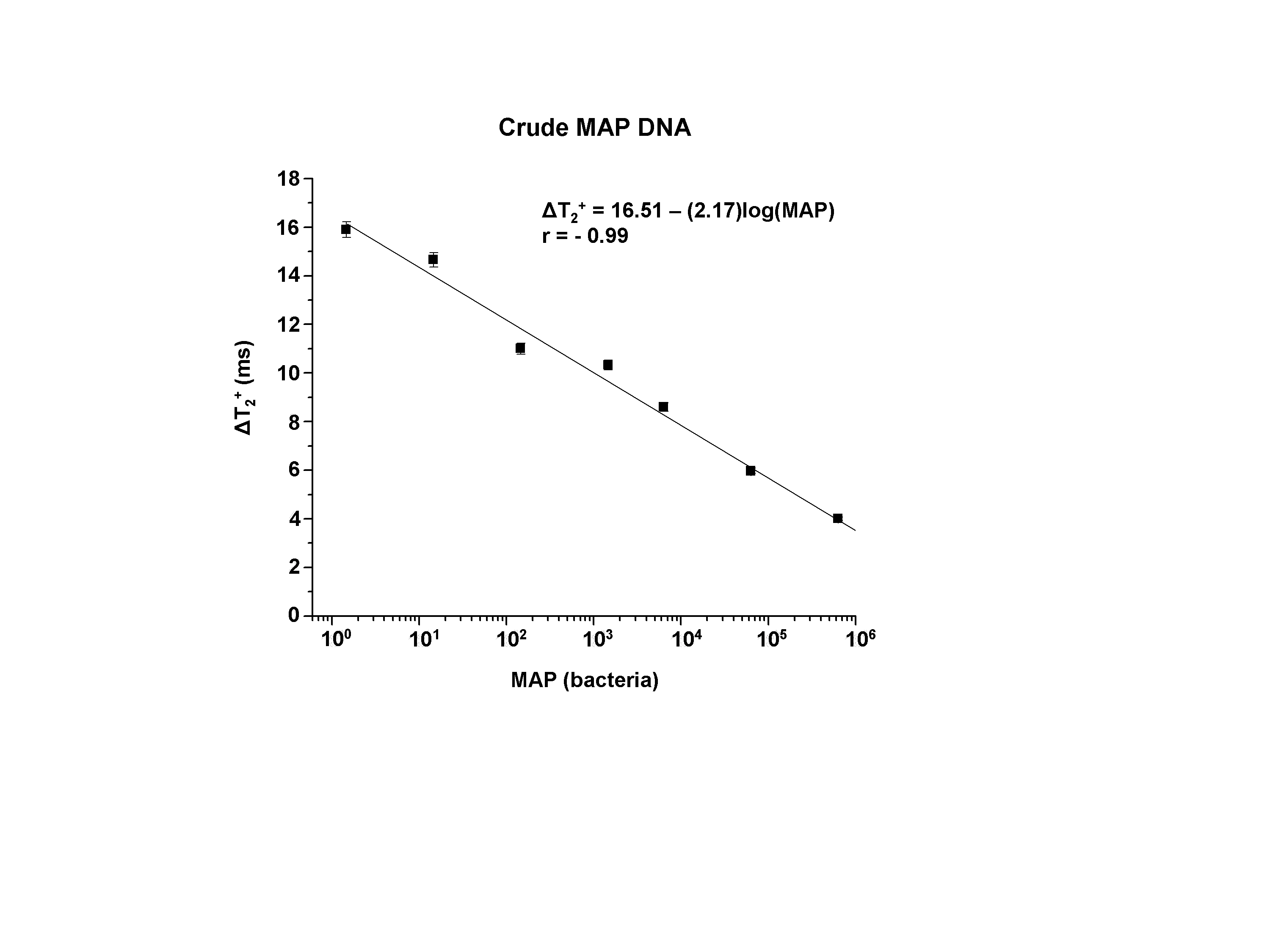

Supplement: Figure S3 — Genome-copy-based quantification of MAP with hMRS and crude extracted DNA. Samples with known amounts of crude DNA from cultured MAP were utilized to correlate the changes in the T2 signal (ΔT2+) and the number of bacteria originally present in the sample, using the MAP’s genome size as a reference. (Means±SE. SE too small to depict in high bacterial levels.) (TIF) [file pone.0035326.s003.tif]
