# Supplementary material for: Rapid and Sensitive Detection of an Intracellular Pathogen in Human Peripheral Leukocytes with Hybridizing Magnetic Relaxation Nanosensors
Source: PLoS One. 2012 Apr 9;7(4):e35326. doi: 10.1371/journal.pone.0035326 (PMC3322147; doi:10.1371/journal.pone.0035326)
Supplement: Table S1 — Spin-spin relaxation times (T2) of crude MAP DNA samples. Serial dilutions of crude MAP DNA samples have been utilized to assess the sensitivity of the hMRS method in minimally processed bacterial cultures. The averages of each independent experiment are listed and the studies’ mean. (PDF) [file pone.0035326.s004.pdf]

| DNA (pg) | T2 (ms) | | | |
|---|---|---|---|---|
| | Experiment A | Experiment B | Experiment C | Mean |
| 0 | 50.93 | 51.67 | 52.32 | 51.64 |
| 0.0076 | 68.25 | 66.84 | 67.90 | 67.66 |
| 0.076 | 65.71 | 66.63 | 66.16 | 66.17 |
| 0.76 | 61.97 | 62.59 | 62.78 | 62.45 |
| 7.6 | 62.31 | 61.68 | 62.11 | 62.03 |
| 33 | 59.98 | 60.33 | 60.52 | 60.28 |
| 76 | 58.26 | 58.84 | 58.49 | 58.53 |
| 330 | 57.74 | 57.36 | 57.67 | 57.59 |
| 760 | 57.14 | 57.29 | 56.96 | 57.13 |
| 3300 | 55.58 | 55.76 | 55.42 | 55.59 |
| 33000 | 53.17 | 53.33 | 53.21 | 53.24 |

**Supplementary Table S1**
