# Supplementary material for: Rapid and Sensitive Detection of an Intracellular Pathogen in Human Peripheral Leukocytes with Hybridizing Magnetic Relaxation Nanosensors
Source: PLoS One. 2012 Apr 9;7(4):e35326. doi: 10.1371/journal.pone.0035326 (PMC3322147; doi:10.1371/journal.pone.0035326)
Supplement: Table S2 — Spin-spin relaxation times (T2) of pure MAP DNA samples with known genome copies. Three independent experiments were performed on pure DNA samples obtained from cultured MAP. Correlation between DNA levels and bacterial populations was achieved by quantifying DNA spectrophotometrically and using the MAP genome size as a reference. (PDF) [file pone.0035326.s005.pdf]

MAP (bacteria)	T2 (ms)			
	Experiment A	Experiment B	Experiment C	Mean
0	51.74	50.86	51.12	51.24
1	156.47	150.34	154.90	153.90
2	138.81	140.50	143.78	141.03
17	139.22	135.36	137.59	137.39
168	120.45	124.82	122.29	122.52
1680	118.72	114.07	116.93	116.57
6710	101.33	100.43	98.55	100.10
16800	94.07	90.75	93.11	92.64
67100	86.52	88.72	89.84	88.36
168000	75.91	76.66	75.10	75.89
671000	75.42	75.06	73.98	74.82

Supplementary Table S2
