# Supplementary material for: Rapid and Sensitive Detection of an Intracellular Pathogen in Human Peripheral Leukocytes with Hybridizing Magnetic Relaxation Nanosensors
Source: PLoS One. 2012 Apr 9;7(4):e35326. doi: 10.1371/journal.pone.0035326 (PMC3322147; doi:10.1371/journal.pone.0035326)
Supplement: Table S3 — Demographics of cultured clinical isolates that were screened with hMRS and nPCR. (CD: Crohn’s disease, IBD: inflammatory bowel disease) (PDF) [file pone.0035326.s006.pdf]

Sample	Sex/Age	Diagnosis	Isolate’s origin	hMRS
WCB1	F/26	CD	Blood	- (0/3)
WCB9	M/22	CD	Blood	- (0/3)
GN2’	M/58	CD	Ileal biopsy	+ (3/3)
GN8’	F/53	CD	Ileal biopsy	- (0/3)
GN2	M/58	CD	Blood	+ (3/3)
GN8	F/53	CD	Blood	+ (3/3)
R16	M/39	Non-IBD	Blood	+ (3/3)
R62B	F/41	CD	Blood	+ (3/3)
R33B	F/33	CD	Blood	+ (3/3)
WC3/1	M/44	CD	Blood	+ (3/3)

Supplementary Table S3
