# Supplementary material for: Rapid and Sensitive Detection of an Intracellular Pathogen in Human Peripheral Leukocytes with Hybridizing Magnetic Relaxation Nanosensors
Source: PLoS One. 2012 Apr 9;7(4):e35326. doi: 10.1371/journal.pone.0035326 (PMC3322147; doi:10.1371/journal.pone.0035326)
Supplement: Table S4 — Clinical data and hMRS results of Cohort 1 blood samples. Samples from Crohn’s disease (CD) patients, healthy individuals or asymptomatic carriers were analyzed by direct nPCR and hMRS. Quantification of the MAP genome copies was achieved in positive (+) samples using a training standard curve for crude extracted MAP DNA. (PDF) [file pone.0035326.s007.pdf]

				hMRS		
**Sample**	**Sex/Age**	**Diagnosis**	**Direct nPCR**	**MAP**	**ΔT2 (ms)**	**MAP (bacteria)**
S1	F/32	CD	+	+ (3/3)	11.2	277
S2	M/44	CD	+	+ (3/3)	2.2	3760791
S3	M/77	CD	+	+ (3/3)	15.3	4
S4	F/70	CD	+	+ (3/3)	6.3	49844
S5	F/58	CD	+	+ (3/3)	4.4	400959
S6	F/20	CD	+	+ (3/3)	10.6	564
S7	M/50	CD	-	- (0/3)	0.1	0
S8	M/32	Healthy	-	- (0/3)	0.1	0
S9	M/30	CD	-	- (0/3)	0.2	0
S10	F/30	CD	-	- (0/3)	0.3	0
S11	M/60	CD	+	+ (3/3)	8.9	3414
S12	F/46	Healthy	+	+ (3/3)	8.7	4002
S13	F/34	CD	+	+ (3/3)	7.2	19826
S14	F/44	CD	+	+ (3/3)	3.1	1449066
S15	M/29	Healthy	-	- (0/3)	1.3	0
S16	M/46	CD	+	+ (3/3)	6.4	47272
S17	M/65	Healthy	+	+ (3/3)	8.4	5348
S18	F/31	CD	+	+ (3/3)	8.7	4220
S19	F/40	CD	+	+ (3/3)	7.6	12826
S20	F/33	CD	+	+ (3/3)	7.3	18214
S21	F/20	CD	+	+ (3/3)	6.5	40754
S22	F/55	Healthy	+	+ (3/3)	9.4	1947
S23	M/44	Healthy	+	+ (3/3)	10.4	633
S24	F/35	CD	+	+ (3/3)	6.5	42972
S25	F/59	CD	+	+ (3/3)	6.3	53682
S26	F/25	Healthy	+	+ (3/3)	11.5	213
S27	F/42	Healthy	+	+ (3/3)	5.7	94133
S28	F/18	CD	+	+ (3/3)	4.0	576373
S29	F/27	CD	+	+ (3/3)	3.9	654529
S30	M/22	Healthy	+	+ (3/3)	12.5	74
S31	F/64	CD	+	+ (3/3)	1.6	7568667
S32	M/19	CD	+	+ (3/3)	1.5	7896377
S33	F/48	Healthy	+	+ (3/3)	12.0	128
S34	F/31	Healthy	+	+ (3/3)	2.1	4408705

Supplementary Table S4
